# The Inactivated ISKNV-I Vaccine Confers Highly Effective Cross-Protection against Epidemic RSIV-I and RSIV-II from Cultured Spotted Sea Bass Lateolabrax maculatus

**DOI:** 10.1128/spectrum.04495-22

**Published:** 2023-05-24

**Authors:** Weixuan Fu, Yong Li, Yuting Fu, Wenfeng Zhang, Panpan Luo, Qianqian Sun, Fangzhao Yu, Shaoping Weng, Wangdong Li, Jianguo He, Chuanfu Dong

**Affiliations:** a State Key Laboratory of Biocontrol (Guangzhou, SYSU)/Southern Marine Science and Engineering Guangdong Laboratory (Zhuhai, SMST-GDL), School of Life Sciences of Sun Yat-sen University, Guangzhou, China; b Institute of Aquatic Economic Animals and Guangdong Province Key Laboratory for Aquatic Economic Animals, Sun Yat-sen University, Guangzhou, People’s Republic of China; c Zhuhai Modern Agriculture Development Center, Zhuhai, China; d School of Marine Sciences, Sun Yat-sen University, Zhuhai, Guangdong, China; University of Prince Edward Island

**Keywords:** *Megalocytivirus*, RSIV genotype, ISKNV genotype, inactivated vaccine, cross-protection, infectious spleen and kidney necrosis virus, red seabream iridovirus

## Abstract

The genus *Megalocytivirus* of the family *Iridoviridae* is composed of two distinct species, namely, infectious spleen and kidney necrosis virus (ISKNV) and scale drop disease virus (SDDV), and both are important causative agents in a variety of bony fish worldwide. Of them, the ISKNV species is subdivided into three genotypes, namely, red seabream iridovirus (RSIV), ISKNV, and turbot reddish body iridovirus (TRBIV), and a further six subgenotypes, RSIV-I, RSIV-II, ISKNV-I, ISKNV-II, TRBIV-I, and TRBIV-II. Commercial vaccines derived from RSIV-I , RSIV-II and ISKNV-I have been available to several fish species. However, studies regarding the cross-protection effect among different genotype or subgenotype isolates have not been fully elucidated. In this study, RSIV-I and RSIV-II were demonstrated as the causative agents in cultured spotted seabass, Lateolabrax maculatus, through serial robust evidence, including cell culture-based viral isolation, whole-genome determination and phylogeny analysis, artificial challenge, histopathology, immunohistochemistry, and immunofluorescence as well as transmission electron microscope observation. Thereafter, a formalin-killed cell (FKC) vaccine generated from an ISKNV-I isolate was prepared to evaluate the protective effects against two spotted seabass original RSIV-I and RSIV-II. The result showed that the ISKNV-I-based FKC vaccine conferred almost complete cross-protection against RSIV-I and RSIV-II as well as ISKNV-I itself. No serotype difference was observed among RSIV-I, RSIV-II, and ISKNV-I. Additionally, the mandarin fish Siniperca chuatsi is proposed as an ideal infection and vaccination fish species for the study of various megalocytiviral isolates.

**IMPORTANCE** Red seabream iridovirus (RSIV) infects a wide mariculture bony fish and has resulted in significant annual economic loss worldwide. Previous studies showed that the phenotypic diversity of infectious RSIV isolates would lead to different virulence characteristics, viral antigenicity, and vaccine efficacy as well as host range. Importantly, it is still doubted whether a universal vaccine could confer the same highly protective effect against various genotypic isolates. Our study here presented enough experimental evidence that a water in oil (w/o) formation of inactivated ISKNV-I vaccine could confer almost complete protection against RSIV-I and RSIV-II as well as ISKNV-I itself. Our study provides valuable data for better understanding the differential infection and immunity among different genotypes of ISKNV and RSIV isolates in the genus *Megalocytivirus*.

## INTRODUCTION

Members of the family *Iridoviridae* are large, icosahedral viruses, containing circular, double-stranded DNA genomes with sizes ranging from 98.6 kb to 212 kb, consisting of 97 to 211 open reading frames (ORFs) and causing severe systemic diseases resulting in significant economic and environmental effects worldwide (1–4). According to the latest release of the International Committee on Taxonomy of Viruses (https://talk.ictvonline.org/taxonomy/), the family *Iridoviridae* is composed of seven genera. Among them, three genera (*Ranavirus*, *Lymphocystivirus*, and *Megalocytivirus*) infect a variety of cold-blooded vertebrates, including bony fish and amphibians, as well as reptiles, whereas the other four genera (*Chloriridovirus*, *Iridovirus*, *Decapodiridovirus*, and *Daphniairidovirus*) infect invertebrates, including insects and crustaceans as well as mollusks. In the past decades, *Megalocytivirus* has attracted increasing interest and concerns due to its causing highly lethal systemic infections in wild and cultured freshwater and brackish and marine bony fish worldwide (5–8). Currently, complete genomic sequences of numerous megalocytiviral isolates have been determined and annotated. According to the conserved core genes, *Megalocytivirus* could be classified into two distinct species, i.e., infectious ISKNV and scale drop disease virus (SDDV) (9, 10). The ISKNV species represents the traditional typical *Megalocytivirus*, consisting of three genotypes or genogroups, namely, red seabream iridovirus (RSIV), ISKNV, and turbot reddish body iridovirus (TRBIV) (6). Recently, the ISKNV species have been further divided into six subgenotypes, including RSIV-I, RSIV-II, ISKNV-I, ISKNV-II, TRBIV-I, and TRBIV-II, respectively (9, 11). Generally, members in ISKNV species, regardless of genotype, have GC content ranging from 53% to 55% and similar genomic sizes of 110,104 bp to 112,636 bp. The general genomic identities among ISKNV species are shared from 93% to 98% (12–14). In contrast, SDDV has a larger genome size (>130 kb) but a much lower genomic identity than ISKNV species (9, 10, 15). Importantly, the very weak antigenic cross-reaction between ISKNV and SDDV results in almost no cross-protection between the inactivated vaccines of RSIV, ISKNV, and SDDV (9, 10, 16).

Among ISKNV species, RSIV was the first defined member, was first isolated from diseased cage-cultured red seabream Pagrus major in Japan in 1990, and has been rapidly spread to over 30 mariculture fish and just a few freshwater fish in Perciformes, Pleuronectiformes, and Tetraodontiformes distributed in the Asia-Pacific area along coastal countries and regions, including China, Japan, South Korea, wide Southeast Asia, as well as in recent South Asia and Central America (6, 14, 17–20). ISKNV, the type species of the genus *Megalocytivirus*, infects a very wide variety of species of freshwater fish and marine-borne fish, as well as a variety of ornamental fish worldwide, since its first occurrence in the freshwater mandarin fish Siniperca chuatsi in 1994 in China (6, 7, 13, 21–26). In contrast to the very wide host ranges of RSIV and ISKNV, TRBIV just infects some marine flatfish and, very rarely, other fish species (11, 27–30). In general, RSIV, ISKNV, and TRBIV represent three genotypes with highly homologous viral properties in ISKNV species. However, phenotypic diversity still exists among the different isolates, even within the same genotype or subgenotype, which might lead to significant differences in virus replication and virulence, as well as host range and vaccine efficacy (8, 29). The inactivated vaccines derived from RSIV-I and ISKNV-I have been developed and licensed for commercial application in Japan and China, respectively (31–33). According to the product descriptions, the former was just licensed to apply in marine-cultured Pagrus major, *Seriola* spp., and Pseudocaranx dentex, and the latter was limited to only freshwater mandarin fish. In addition, an RSIV-II-based inactivated vaccine (Aquavac IridoV) was also in development by MSD Animal Health. Given that all these vaccines are only commercially available in several countries, studies concerning cross-protection among different vaccine candidates have rarely been performed. However, an application of the commercial oil-based RSIV-II vaccine in Vietnamese Asian seabass Lates calcarifer (also known as barramundi) farm showed a quite low efficiency against the natural outbreak of ISKNV-II (34). Generally, all this information suggests that the differential phenotypes among different RSIV and ISKNV isolates may influence the vaccine cross-protective effects, and there is no universal vaccine that can be taken for granted to confer the same highly effective protection upon various RSIV and ISKNV infections. In this study, both RISV-I and RISV-II were confirmed as the etiologic agents in the cultured spotted sea bass Lateolabrax maculatus. The pathogenic properties were first investigated in detail in mandarin fish and spotted seabass. The cross-protection of the ISKNV-I-based vaccine against both types of RSIVs was also fully evaluated in mandarin fish as well as in spotted seabass.

## RESULTS

### Isolation and identification of RISV-I and RISV-II from diseased spotted seabass.

**FIG 1:**
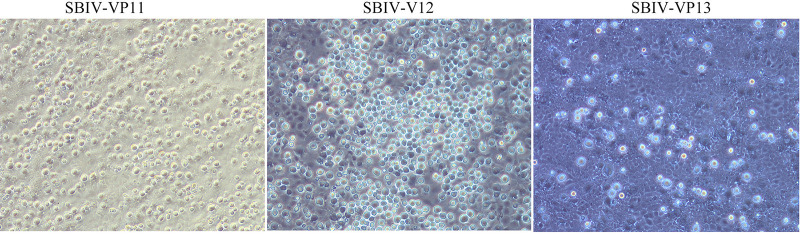
Isolation of spotted seabass iridovirus (SBIV) using MFF-1 cells. Three viruses were isolated from three batches of diseased spotted seabass samples and designated SBIV-VP11, -V12, and -VP13, respectively.

**FIG 2:**
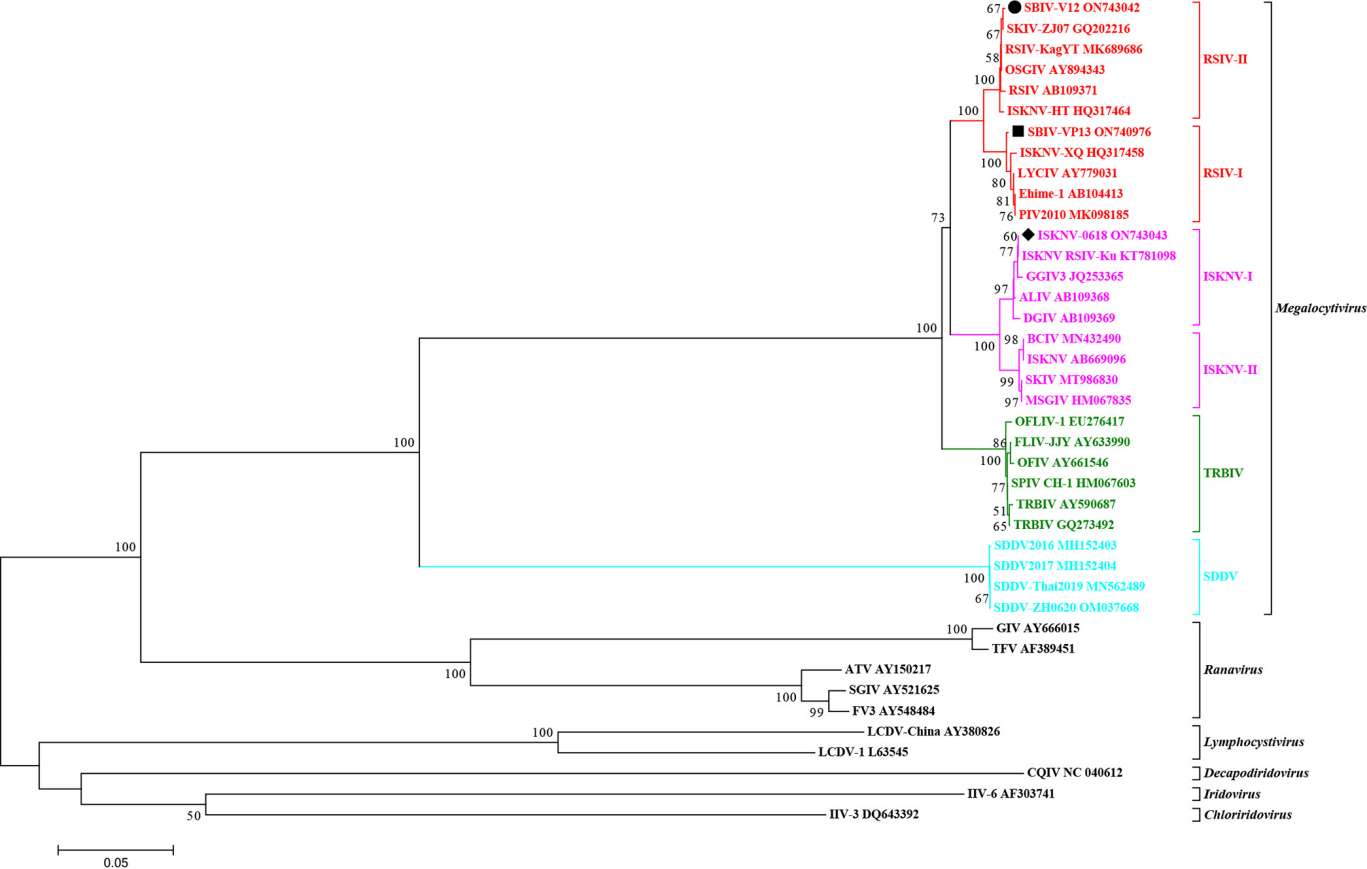
Phylogenetic tree of members within *Iridoviridae* based on the *mcp* gene. SBIV-V12, SBIV-VP13, and ISKNV-0618 are clustered into genotypes of RSIV-II, RSIV-I, and ISKNV-I, respectively.

Diseased fish samples from three mass mortality events of spotted seabass were used to isolate viruses, and three viruses were isolated. All viruses induced the typical cytopathic effects (CPEs) characterized by numerous enlarged rounding cells (Fig. 1), which are highly consistent with the general typical CPEs induced by other megalocytiviral isolates (35–37). Thus, these viruses were predicted as megalocytiviruses and designated SBIV-VP11, -V12, and -VP13, respectively. The complete sequences of the *mcp* gene of these three SBIVs were cloned. The results showed that SBIV-VP11 and SBIV-V12 share 100% identity of the *mcp* sequence, whereas SBIV-VP13 has a distinct *mcp* sequence. Based on the *mcp* sequence, phylogenic analysis showed that SBIV-V12 and SBIV-VP13 were clustered into RISV-II and RISV-I, respectively (Fig. 2). Additionally, the mandarin fish original ISKNV-0618 was clustered in ISKNV-I, and the RISV vaccine strain Ehime-1 in Japan was grouped into RISV-I (Fig. 2).

To better understand the genomic properties of the isolated SBIVs, whole-genome sequences of SBIV-V12 and SBIV-VP13 were determined, annotated, and deposited in GenBank with accession numbers ON743042 and ON740976, respectively. The results showed that the genomes of SBIV-VP13 and SBIV-V12 are composed of 112,158 bp and 111,876 bp and encode 119 and 118 putative ORFs, respectively. The GC content of both genomes is 53%, which is highly consistent with those of several other sequenced ISKNV, RSIV, RBIV, orange-spotted grouper iridovirus (OSGIV), and large yellow croaker iridovirus (LYCIV) isolates (12–14, 38, 39).

**FIG 3:**
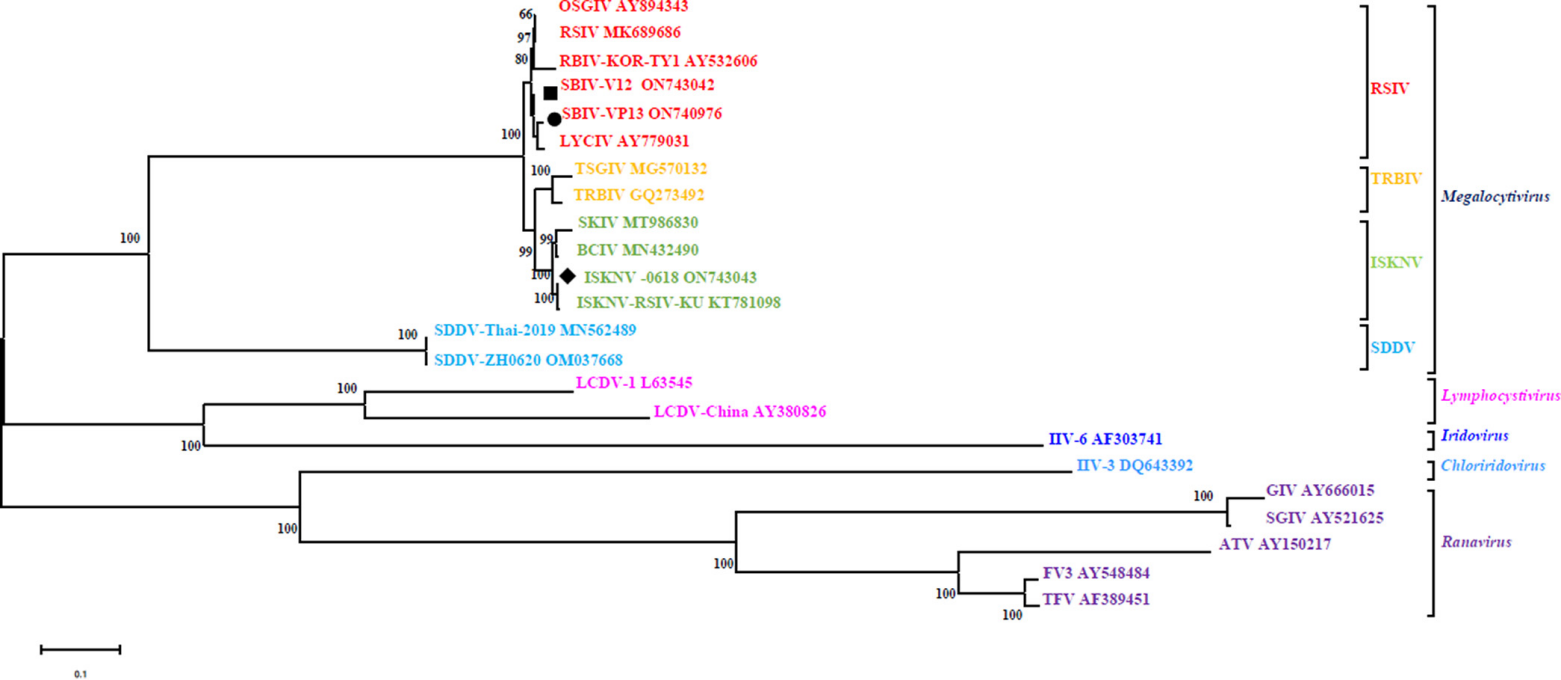
Phylogenetic tree of 26 iridoviral core genes sequences from 23 completely sequenced iridoviruses. The spotted seabass original SBIV-V12 and SBIV-VP13 were clustered into the RSIV clade, whereas ISKNV-0618 is clustered into the ISKNV clade.

The conserved regions of 26 iridoviral core genes sequences were compared by Clustal X 2.0 and gBlock tool, and the best tree-building pattern was found to be GTR+G+I using “Find Best DNA/Protein Models” in MEGA 7.0 software. The maximum-likelihood (ML) phylogenetic tree was constructed with this model, and 1,000 replicates were performed. The phylogenetic tree showed that 14 megalocytiviral isolates can be classified into 4 groups, namely, RSIV, ISKNV, TRBIV, and SDDV. Among them, RSIV, ISKNV, and TRBIV consist of the most closed phylogenetic cluster, whereas SDDV represents another distant phylogenetic cluster. Further, the spotted seabass original SBIV-V12 and -VP13 were clustered into the RSIV clade, whereas ISKNV-0618 was clustered into the ISKNV clade (Fig. 3).

### Both SBIVs showed high lethality in mandarin fish with the featured histopathology.

**FIG 4:**
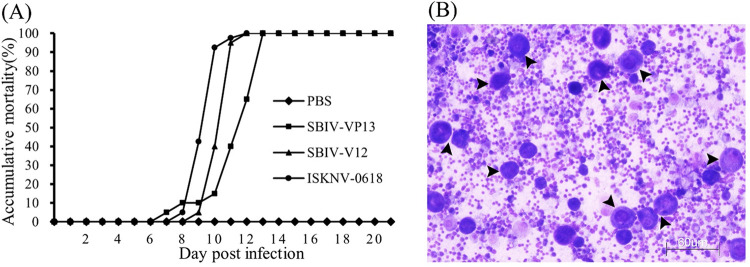
Infectivity of SBIV-SBIV-V12 and -VP13, and ISKNV-0618 on mandarin fish. (A) Mortality curves of mandarin fish infected with SBIV-VP13, SBIV-V12, and ISKNV-0618. (B) Numerous abnormally enlarged splenic cells of diseased mandarin fish upon challenge by SBIV-VP13. Arrows indicate the abnormally enlarged splenic cells by imprint analysis of the infected spleen.

SBIV-V12 and SBIV-VP13, as well as ISKNV-0618, were used to infect mandarin fish with the same infection dose. As a result, all viruses caused 100% mortality (*n* = 20) to mandarin fish within 14 days (Fig. 4A). The onsets of peak mortalities of the three viruses began with ISKNV-0618, followed by SBIV-V12 and then SBIV-VP13. All diseased fish showed similar clinical symptoms characterized by vomiting, lethargy, pale gills, and abnormal swimming. After autopsy, the spleen and kidneys were observed to be enlarged and had become dark red, and the liver became pale with hemorrhagic spots. By imprint analysis of the infected spleen, numerous abnormally enlarged basophilic cells could be clearly observed (Fig. 4B), which was highly consistent with previous observations (36, 37).

**FIG 5:**
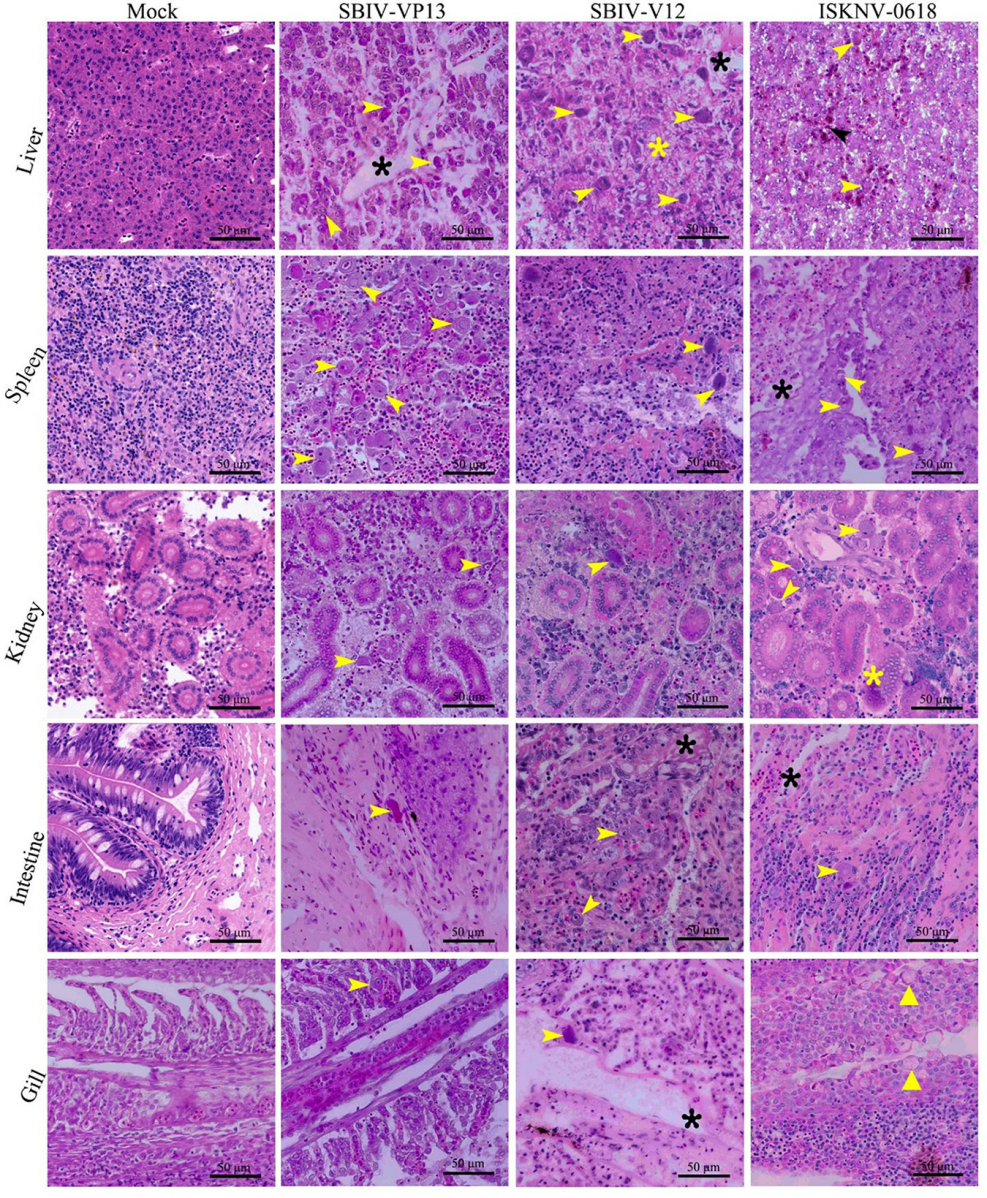
Histopathology of SBIV-V12-, SBIV-VP13-, and ISKNV-0618-infected mandarin fish. Yellow arrows indicate the typical enlarged cells, black asterisks indicate the focal necrosis of tissue, yellow asterisks indicate vacuolation cells, black arrowheads indicate the phenomenon of erythrocyte infiltration, and yellow triangles arrowheads indicate edema of secondary gill filaments.

**FIG 6:**
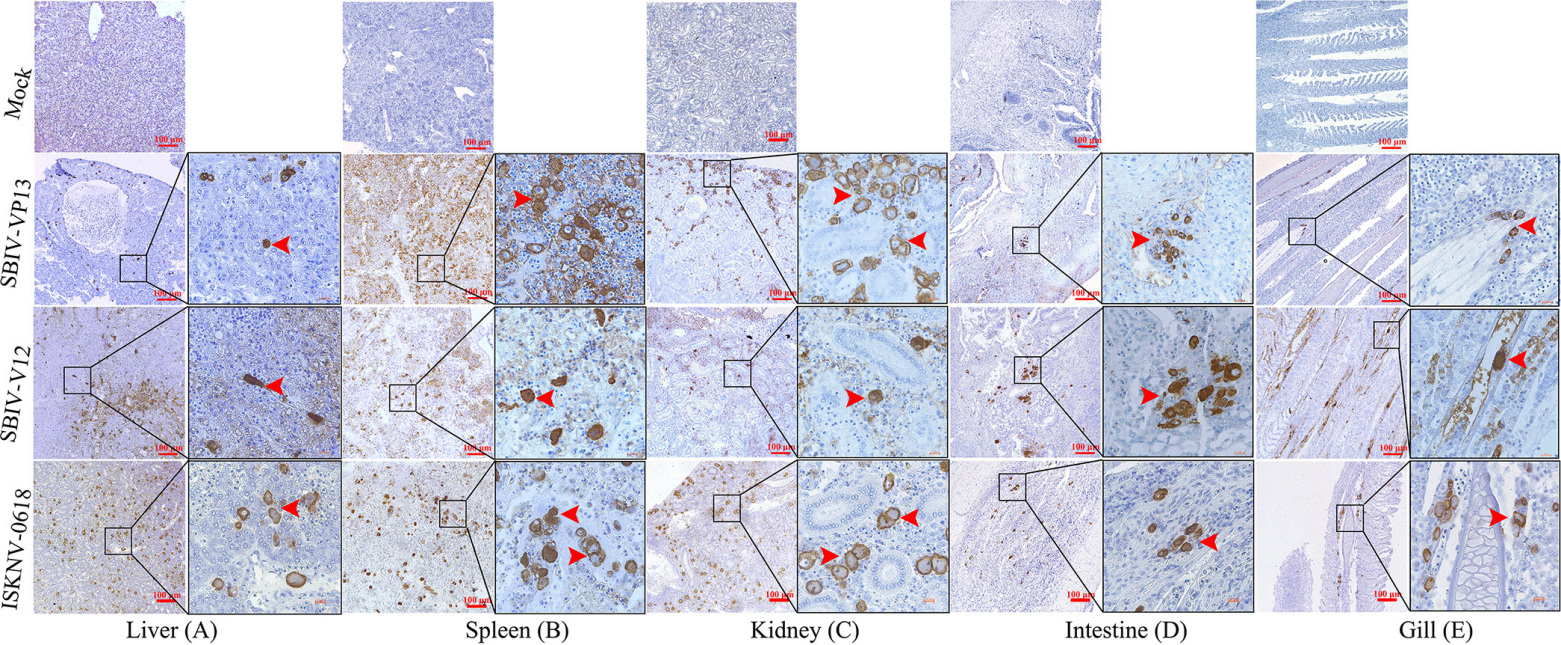
Immunohistochemical assay (IHC) of SBIV-V12-, SBIV-VP13-, and ISKNV-0618-infected mandarin fish. Numerous enlarged cells from infected liver, spleen, kidney, intestine, and gill were well recognized by anti-ISKNV-VP23 polyclonal antibody (pAb) and were visualized as brown. No signal was observed in uninfected healthy mandarin fish. Arrows indicate the characteristic abnormally enlarged cells.

**FIG 7:**
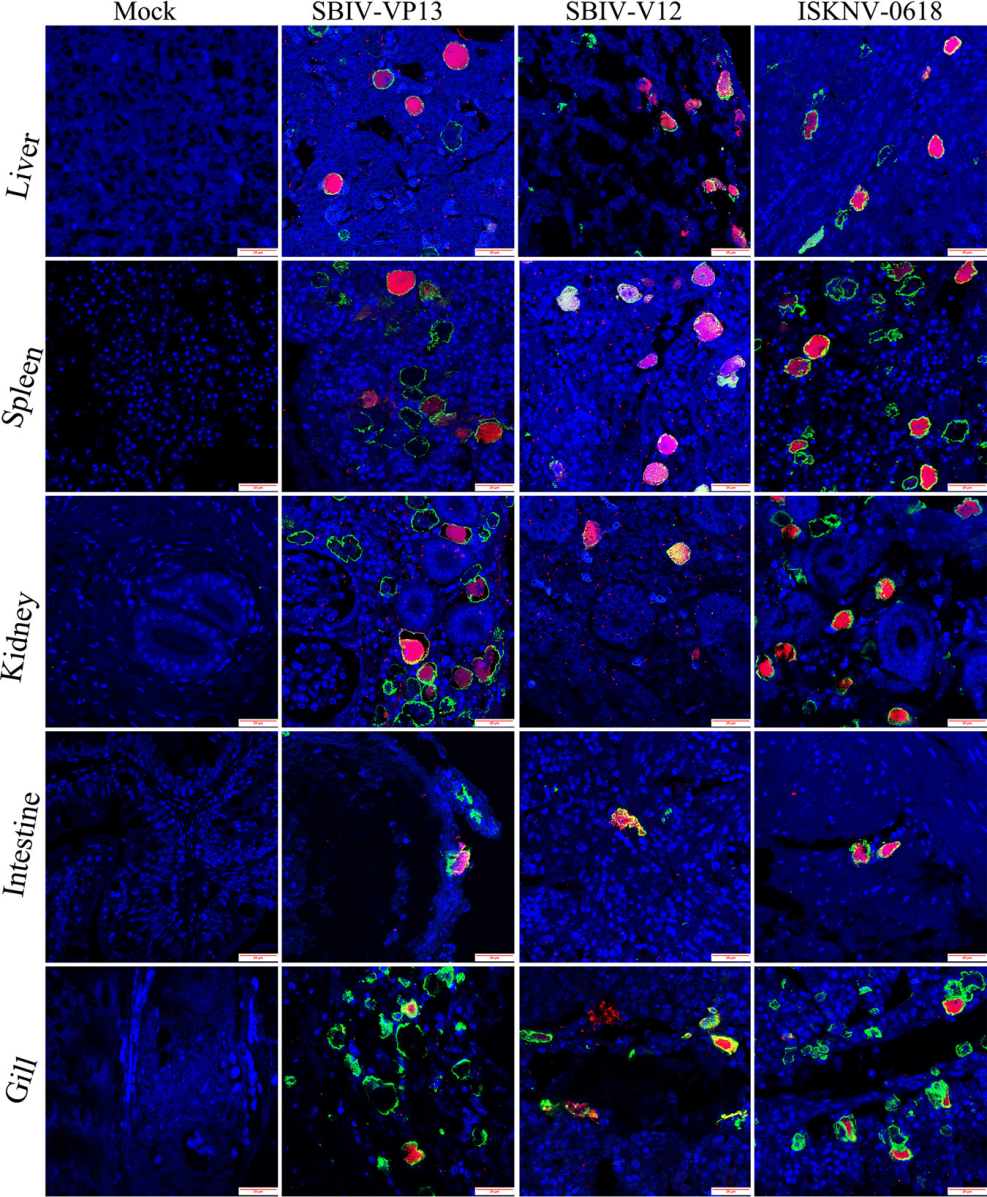
Double-stained immunofluorescence examination of SBIV-V12-, SBIV-VP13-, and ISKNV-0618-infected mandarin fish. Green fluorescence is associated with anti-nonstructural ISKNV-VP23 MAb, and red fluorescence is associated with antistructural ISKNV-VP101 pAb.

**FIG 8:**
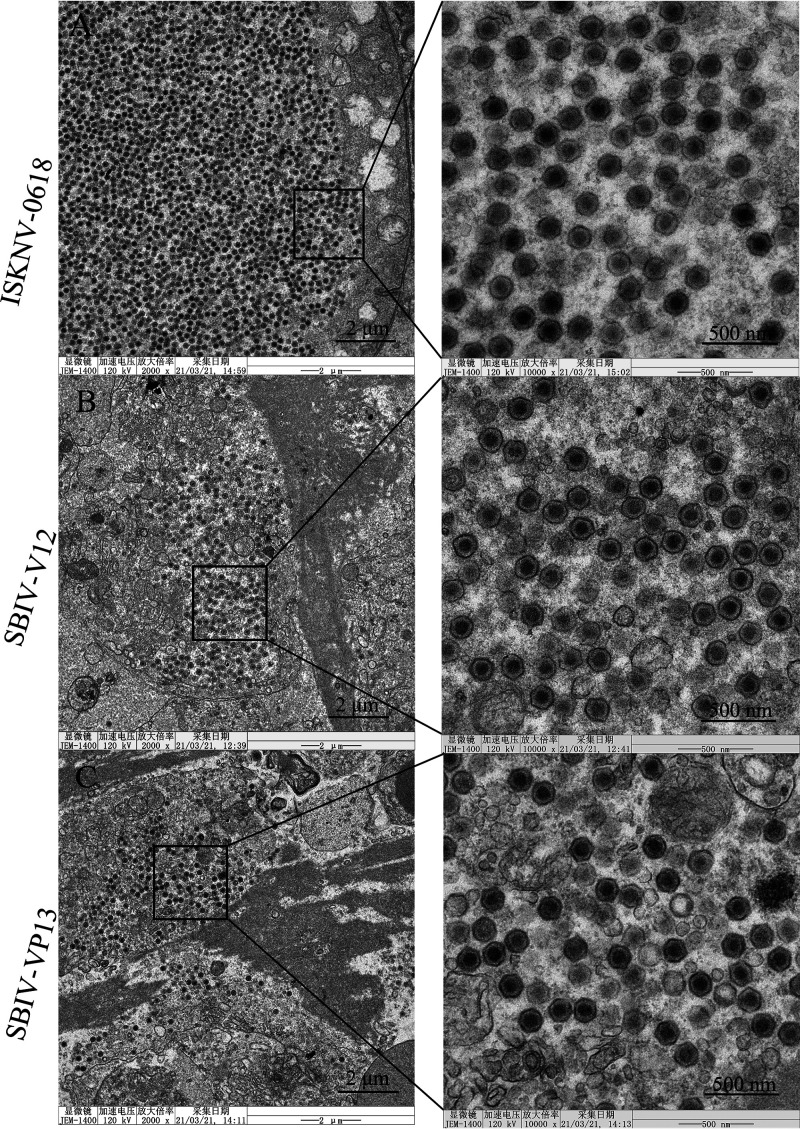
Transmission electron micrograph of the spleen tissue from infected mandarin fish. A large number of virus particles were observed regardless of infection with ISKNV-0618 (A), SBIV-V12 (B), or SBIV-VP13 (C).

Histopathological examination showed that significant histopathological changes were clearly observed in infected liver, spleen, kidney, intestines, and gill (Fig. 5). The most featured histopathology was characterized by numerous abnormally enlarged cells in all the infected tissues. Additionally, other general histopathological signs, such as tissue necrosis foci, cell vacuolization, erythrocyte infiltration, and gill edema, could also be observed. The virus infection was further confirmed by immunohistochemistry (IHC) and immunofluorescence assay (IFA) examination. Based on anti-ISKNV-VP23 pAb, IHC showed that numerous brown positive viral signals were observed in all investigated tissues, including liver, spleen, kidney, intestines, and gill (Fig. 6). Based on a double-staining approach by anti-ISKNV-VP23 monoclonal antibody (MAb) combination with anti-ISKNV-VP101 polyclonal antibody (pAb), IFA showed that both fluorescence signals were clearly observed in all investigated tissues (Fig. 7). Transmission electron microscopy (TEM) analysis showed that a large number of viral particles were observed in the infected spleen tissues (Fig. 8). Based on the above-described results of mortality, histopathology, IHC, IFA, and TEM, our study clearly demonstrated that spotted seabass original SBIV-V12 and SBIV-VP13, as well as an early mandarin fish original ISKNV-0618, are highly virulent to mandarin fish.

### The inactivated ISKNV-I vaccine conferred the same high protection against SBIV-V12 and -VP13, as well as ISKNV-I itself.

**FIG 9:**
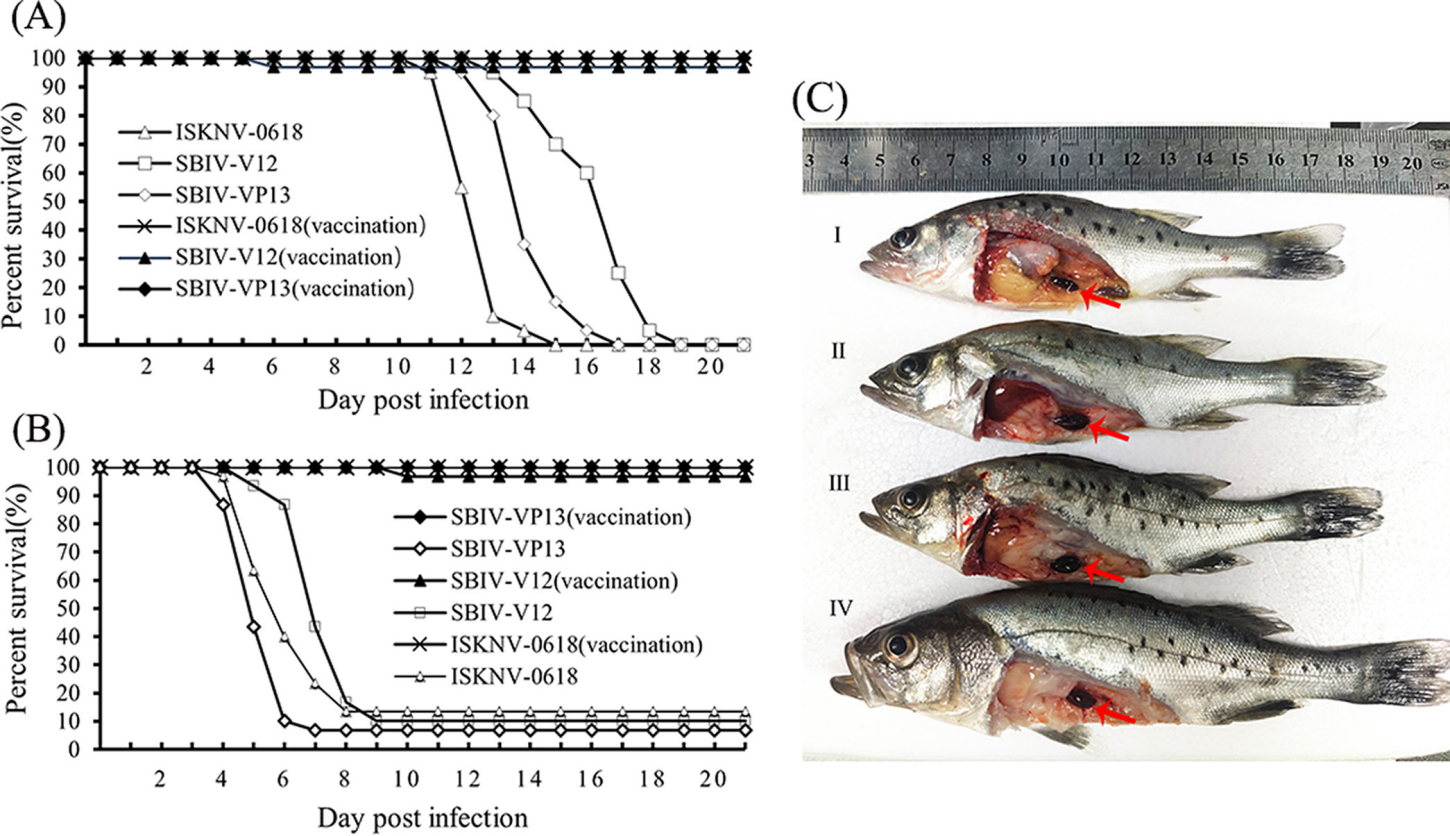
Efficacies of the inactivated ISKNV-0618 vaccine against SBIV and ISKNV. (A) Vaccine efficacies in mandarin fish vaccination model. (B) Vaccine efficacies in spotted seabass. (C) Clinical symptom of diseased spotted seabass from control group upon SBIV challenge. Arrows indicate enlarged spleen.

**FIG 10:**
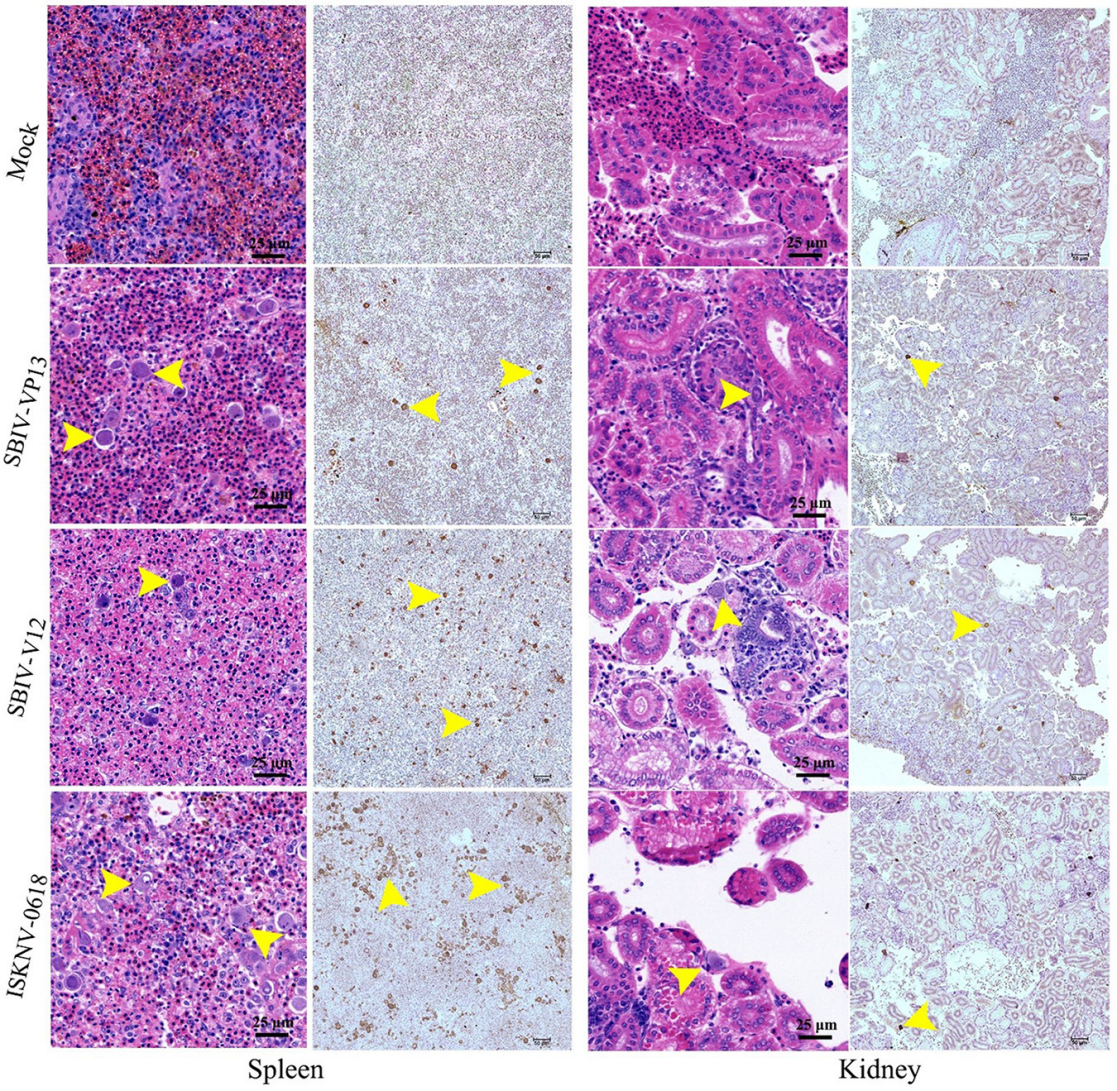
Histopathological and immunohistochemical examinations of the spleen and kidney tissues from infected spotted seabass. Numerous abnormally enlarged cells were observed by H&E staining and were visualized as brown signals (arrows) by anti-ISKNV-VP23 pAb-based IHC.

**FIG 11:**
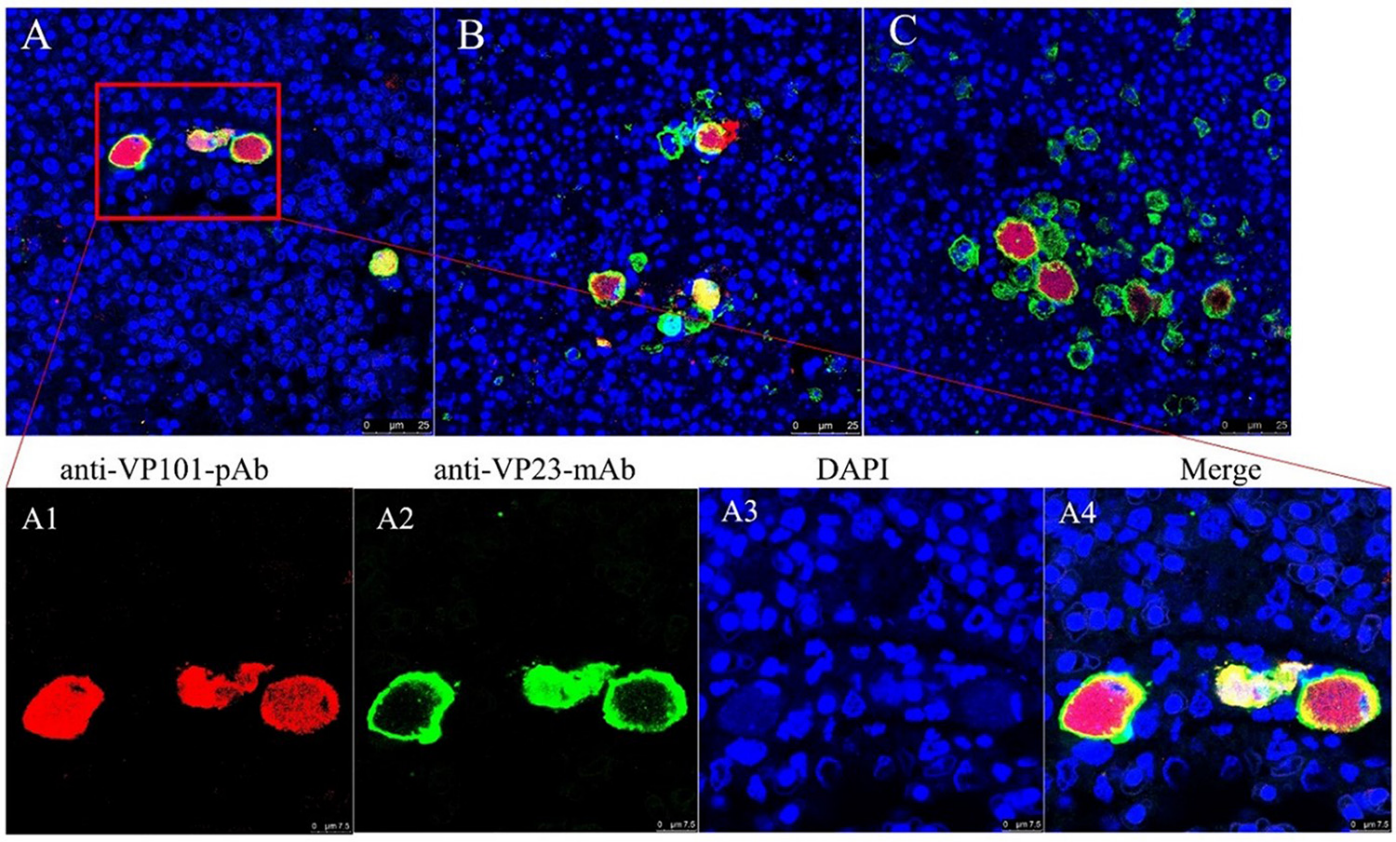
Double-stained immunofluorescence examination of SBIV- and ISKNV-infected spotted seabass. Green fluorescence is associated with anti-nonstructural ISKNV-VP23 MAb, and red fluorescence is associated with anti-structural ISKNV-VP101 pAb. (A to C) Infections with SBIV-VP13, SBIV-V12, and ISKNV-0618, respectively.

Mandarin fish and spotted seabass were immunized by intraperitoneal (i.p.) injection of ISKNV-0618-based formalin-killed cell (FKC) vaccine. The immunized fish were challenged with SBIV-V12, SBIV-VP13, and ISKNV-0618, respectively, at 21 days postvaccination (dpv). Equal unimmunized spotted seabass or mandarin fish were challenged with three viruses as controls, respectively. As a result, mortality of the unimmunized mandarin fish occurred at 11 days postchallenge (dpc), and the cumulative mortalities (*n* = 30) upon SBIV-VP13 and -V12 and ISKNV-0618 challenge within 20 days were all 100% (Fig. 9A). In contrast, the survival rates of three immunized groups were 100% (30/30), 96.7% (29/30), and 100% (30/30), respectively. Thus, the calculated relative percentage survival (RPS) rates of the immunized mandarin fish were 100%, 96.7%, and 100%, respectively. As for spotted seabass, the unimmunized fish began to die at 4 dpi, and the cumulative mortality against SBIV-VP13 and -V12, and ISKNV-0618 within 20 dpc was 93.3% (28/30), 90% (27/30), and 86.7% (26/30), respectively. In contrast, the corresponding survival rates of the three immunized groups were 100% (30/30), 96.7% (29/30), and 100% (30/30), respectively. The calculated RPS rates in spotted seabass were 100%, 96.3%, and 100%, respectively. The histopathology of diseased spotted seabass was also investigated. The result showed that SBIV-VP13 and SBIV-V12, as well as ISKNV-0618, could cause a similar characteristic histopathology featuring numerous abnormally enlarged cells in the main target spleen and kidney (Fig. 10), which was further confirmed by IHC (Fig. 10) and IFA (Fig. 11).

## DISCUSSION

During 2020, diseased spotted seabass from three mass mortality events in Zhuhai, Guangdong, the most important breeding area for commercial spotted seabass in China, were collected by our laboratory for pathogen identification. From the featured clinical syndromes described by the local famers, RSIV and ISKNV were proposed as the causative agents for these mortality events. Thus, the tissue homogenates from these diseased fish samples were directly used to inoculate into mandarin fish fry (MFF-1) cells for virus isolation. As a result, three viruses with similar CPEs characterized by numerous bulging and rounding MFF-1 cells were isolated (Fig. 1). Using a universal primer set (37), the complete *mcp* genes were cloned and determined. SBIV-VP11 and SBIV-V12 have complete *mcp* sequence identities and were clustered into RSIV genotype II (RSIV-II), whereas SBIV-VP13 has a distinct *mcp* identity and was clustered into RSIV genotype I (RSIV-I). Thereafter, whole-genome sequences of SBIV-V12 and -VP13 were determined and annotated. Regardless of their genomic content and encoding genes, V12 and VP13 have high similarities. Eaton et al. proposed 26 common genes as the core iridoviral mark to reannotate iridoviral members (38). The tandem 26 core iridoviral genes were used to build a phylogenetic tree (Fig. 3); however, the ML phylogenetic tree is rough, rather than fine, in distinguishing these members of the same ISKNV species. Generally, RSIV-II is always the prominent strain in various fish species in mainland China (20, 37, 39), whereas RSIV-I has just been previously documented in mariculture in the large yellow croaker, Larimichthys crocea; silver pomfret; Pampus argenteus (40–42); and freshwater spotted mandarin fish, Siniperca scherzeri (43). It is unusual to isolate both RSIV-I and RSIV-II in the same fish species. Our study demonstrated that both RSIV-I and RSIV-II were isolated from the mass mortalities of spotted seabass in the same breeding area during the same breeding annual, suggesting that there are multiple pathogenic sources for the cultured spotted sea bass in Zhuhai, Guangdong. On the other hand, spotted seabass is a highly sensitive host fish species for both RSIV-I and RSIV-II. In view of the limited strains collected in this study, it is still difficult to determine which genotype RSIV is the dominant epidemic strain in spotted seabass. However, the first matter of importance is to find effective treatments to control them.

As a highly sensitive fish species for both ISKNV and RSIV (20, 36, 37), mandarin fish was used to test the lethality of two SBIVs as well as the ISKNV-I strain 0618 under the same infection dose. The result showed that both SBIVs and ISKNV-0618 were 100% lethal to mandarin fish. The lethal infections were further confirmed by histopathology, IHC, and IFA, as well as TEM. In addition, from the mortality curves, the mortality peak of ISKNV-0618 was about 2 to 3 days earlier than those of two SBIV isolates, which may be due to the phenotype diversity among different isolates as previously reported (8). In Shinmoto et al.’s study, although RSIV isolates U-1, U-6, and KST-Y-1 have the complete *mcp* sequence identity and belong to the same subgenotype, these isolates still exhibited significantly different replication rates and virulence, as well as vaccine efficacies. Of note, the RPS rates of red seabream juveniles immunized with U-6, U-1, and KST-Y-1 strains were 69, 45, and 11%, respectively, against challenges with the same U-6 strain (8), indicating very significant differences in vaccine efficacy among different isolates. Because of the lack of efficacy resulting from these vaccines against their parental strains, it is difficult to determine whether the poor efficacy of these vaccines is due to the poor antigenicity of the vaccine strain itself or the poor protection that just occurred against heterologous strains. It was reported that the RSIV-I genotype of the Ehime-1-based inactivated vaccine has been developed and licensed in Japan and was commercially available in red seabream as well as for fish belonging to the genus *Seriola*, striped jack (Pseudocaranx dentex), Malabar grouper (Epinephelus malabaricus), and orange-spotted grouper (Epinephelus coioides) but not suitable for fish species in the genus *Oplegnathus* because of the high susceptibility of these species to RSIV infection (6). The Ehime-1-based injectable vaccine has been also licensed in mainland China. According to the product description, this vaccine was just limited to P. major, *Seriola* spp., and P. dentex. Thus, it is very difficult to obtain this vaccine for an efficacy evaluation in other fish species, especially in the highly susceptible mandarin fish. Based on a highly permissive MFF-1 cell line (36), an oil-based formalin-killed ISKNV-I vaccine has been developed and showed >95% absolute protection effect in mandarin fish regardless of whether under a laboratory or field trial test (20, 32). The improved oil-based ISKNV-I (NH0618 strain) vaccine was officially licensed by China’s Ministry of Agriculture and Rural Affairs at the end of 2019 and is now commercially available for mandarin fish. The major concern is whether the ISKNV-I vaccine can expand to protect a broader fish species against the heterologous RSIV and ISKNV pathogenic strain. In this study, both spotted seabass original RSIV-I and RSIV-II isolates experimentally evidenced 100% lethality in mandarin fish. Thereafter, the oil-based ISKNV-I FKC vaccine was prepared to assess the protection effects in mandarin fish as well as in spotted seabass. As a result, the RPS rates (*n* = 30) against RSIV-I and RSIV-II, as well as ISKNV-I itself, in mandarin fish and spotted seabass are 100%, 96.7%, and 100% versus 100%, 96.3%, and 100% (Fig. 9A and B). The RPS rates between the two fish species showed no significant difference. Histopathology, as well as IHC and IFA, demonstrated that three viruses could cause high lethality to unimmunized spotted seabass, with the same featured clinical signs and histopathology as observed in infected mandarin fish, indicating that all spotted seabass died of RSIV and ISKNV infection. It is worth noting that mandarin fish original ISKNV-0618 showed more virulence to mandarin fish than two SBIV isolates; however, the RSIV-I type of SBIV-VP13 showed more virulence to spotted seabass than ISKNV-0618, indicating that the pathogenicity of different isolates may be associated with a certain host specificity, which may explain why RSIV, ISKNV, and TRBIV have individual sensitive host fish.

The significant difference was that all three viruses are 100% lethal to mandarin fish with a larger body weight (about 50 g/fish) but about 90% lethal to spotted seabass with a smaller body weight (about 30 g/fish). As a matter of fact, ISKNV- and RSIV-associated outbreak occurs throughout the whole breeding process in mandarin fish, from fingerling to mature fish. There are always two ISKNV and RSIV outbreak peaks during mandarin fish breeding. One occurs at the fingerling stage when is the turn of spring to summer, with water temperature slowly rising; another occurs at the large-size growing-out stage when is the turn of summer to autumn, with water temperature slowly dropping. In contrast, RSIV-associated mortality of spotted seabass was monitored commonly in the fingerling stage but rarely in the large-sized growing-out stage. Generally, mandarin fish is more sensitive to various ISKNV and RSIV strains than spotted seabass. Thus, it is not unexpected that the ISKNV vaccine with almost perfect protection in mandarin fish confers the same high efficacy in spotted seabass. A clinical field trial test of the RSIV-II-derived oil-based inactivated vaccine in Vietnamese Asian seabass showed a low efficacy against the natural outbreak of ISKNV-II (34). Due to the very limited available information, it is difficult to judge what is the truth of the vaccination failure in Asian seabass. Another experimental study showed that the survival of TRBIV-like founder iridovirus (FLIV)-infected rock bream conferred no cross-protection against the rechallenge with rock bream iridovirus (RBIV), an RSIV-II isolate. The authors thus concluded that the genotypic and antigenic differences between FILV and RBIV may influence their cross-protection (44). However, since the report also lacked data on the immune protection of the live FLIV nonlethal infection against its own parental virus as a positive control, this was not a strict vaccine evaluation study, and the conclusion of the so-called no-cross-protection effect was not rigorous. In our recent study, an oil-based inactivated ISKNV-I and SDDV bivalent vaccine was developed and demonstrated to effectively protect Asian seabass against challenges with ISKNV-I, ISKNV-II, RSIV-II, and SDDV (45). All of these data indicate that the oil-based inactivated ISKNV-I vaccine is a promising universal vaccine to protect sensitive fish from infection with various ISKNVs and RSIVs, i.e., ISKNV-I and -II and RSIV-I and -II. Since the TRBIV genotype virus was not preserved in our laboratory, the cross-protective effect of the ISKNV-I vaccine against TRBIV is still unclear. Taken together, this study provided robust evidence that the inactivated ISKNV-I vaccine could almost fully protect spotted seabass against RSIV-I and RSIV-II, as well as ISKNV-I itself.

## MATERIALS AND METHODS

### Fish sampling and virus isolation.

Fish samples were obtained from three mass mortalities of pond-cultured spotted seabass in Zhuhai, Guangdong Province, China. The diseased fish ranged from around 3.5 g to 50 g/fish, with cumulative mortalities calculated by the local fish farmers to be ~70% to 90% within 2 weeks. During outbreaks, fish exhibited the typical *Megalocytivirus*-associated clinical symptoms characterized by lethargy, pale gill, body darkening, and abnormal swimming as previously described elsewhere (7, 34). Fish samples were dissected, and the mixture of spleen and kidney was homogenized with nine volumes of sterilized phosphate-buffered saline (PBS; pH 7.4). After centrifugation at 7,500 × *g* at 4°C for 10 min, the supernatant was collected and filtrated through a 0.22-μm filter membrane (Millipore, USA). The mandarin fish fry (MFF-1) cell line was grown in ambient air with 5% CO_2_ at 26°C with Dulbecco modified Eagle medium (DMEM) supplemented with 10% fetal bovine serum (FBS) (Gibco, Invitrogen, USA) (36). For virus isolation, 50 μL of each sample supernatant was inoculated into the monolayer MFF-1 cells growing in a 75-cm^2^ flask. The infected cell was observed daily until cytopathic effect (CPE) appeared. The isolated virus was kept at −80°C until use. All animal experimental procedures were approved by the Institutional Animal Care and Use Committee, Sun Yat-sen University (SYSU-IACUC-2022-001149).

### Genome determination and annotation.

Briefly, virus-infected MFF-1 cells were collected using the FastPure Cell/Tissue DNA isolation minikit (Vazyme, China) to prepare genomic DNA as follows: 5 × 10^6^ cells were collected and centrifuged at 400 × *g* for 5 min, and the supernatant was discarded. We added 220 μL PBS, 10 μL RNase solution, and 20 μL proteinase K to the samples and resuspended the cells. They were allowed to stand at room temperature for 15 min or more. We added 250 μL GB buffer to the cell resuspension and vortexed for 15 to 30 min. We added 180 μL anhydrous ethanol to the digestion solution and vortexed for 15 to 20 s. The mixture was transferred to the adsorption column and centrifuged at 13,400 × *g* for 1 min. We discarded the filtrate, added 500 μL washing buffer A to the column, and centrifuged at 13,400 × *g* for 1 min. We discarded the filtrate, added 650 μL of washing buffer B to the column, and centrifuged at 13,400 × *g* for 1 min. We repeated the previous step once, discarded the filtrate, and centrifuged the empty tube at 13,400 × *g* for 2 min. We placed the column in a new 1.5-mL centrifuge tube. We added 30 to 100 μL of elution buffer prewarmed to 70°C and left it at room temperature for 3 min. We centrifuged at 13,400 × *g* for 1 min and kept the harvested DNA at −20°C. We added 1 μg of viral DNA to the construct library. Genomic DNA was processed into 350-bp DNA fragments using the NEBNext Ultra DNA library preparation kit (NEB, Ipswich, MA, USA) according to the manufacturer's recommendations. These tiny fragments were then end polished, A-tailed, and adapter ligated. Finally, the libraries were analyzed for quality control based on quantitative PCR (qPCR) absolute quantification. Qualified libraries were sequenced using Illumina NovaSeq PE150 at Beijing Novogene Bioinformatics Technology Co., Ltd. (Beijing, China). The obtained raw data were filtered to obtain clean data and then subjected to *de novo* assembly using MEGAHIT. The assembly results were integrated using SnapGene software. Viral genomes were submitted to ORFfinder (https://www.ncbi.nlm.nih.gov/orffinder/) and the Fgenesv0 program (http://www.softberry.com/berry.phtml?topic=virus0&group=programs&subgroup=gfindv) to predict all potential ORFs. The gene functions and structures were predicted based on BLASTp searches against NCBI and SMART (http://smart.embl-heidelberg.de/). The annotation results of viral genomes were converted using GB2sequin (46) for submission to the NCBI network service.

### Phylogenetic analysis.

The viral genomic DNAs were used as the templates to amplify the *mcp* gene by a set of universal primers (MCP-F, 5′-TCACCGTCATCATGTCTGC-3′, and MCP-R, 5′-AGACACACGGGGCAATC-3′) as in our previous report (37). The PCR products were cloned into the pMD-19T vector and transformed into Escherichia coli DH5α cells. The positive clones were selected for sequencing. The complete sequence of the *mcp* gene was subjected to homological analysis with NCBI’s BLAST program (https://blast.ncbi.nlm.nih.gov/Blast.cgi). Sequences were aligned using ClustalX, and phylogenetic trees were constructed using the neighbor-joining algorithm of MEGA 6.0 software with 1,000 bootstrapped replicates. In addition, an ML phylogenetic tree was constructed on the basis of 26 core iridoviral genes (38).

### Experimental challenge to mandarin fish.

Mandarin fish juveniles with an average body weight of about 30 g were obtained from a local fish farm and used for the experimental challenge. The fish were kept for acclimatization for 10 days before challenge and were detected as virus free by conventional PCR (9). Fish were divided into 4 groups consisting of 20 individuals each in a 50-L glass tank with an air-pumped circulating water system. The water temperature was kept at 27°C, and the fish were fed with bait fish once a day. Spotted seabass original SBIV-VP13 and SBIV-V12 and mandarin fish original ISKNV-0618 were propagated in MFF-1 cells as previously described (36). Fish in three groups were injected intraperitoneally with 0.1 mL of 10^5.0^ 50% tissue culture infective dose (TCID_50_)/0.1 mL of SBIV-V12 and -VP13 and ISKNV-0618, respectively. Twenty fish injected intraperitoneally with 0.1 mL of sterilized PBS were set as a control. Mortality was monitored daily for 21 days.

### Vaccine preparation, immunization, and challenge.

ISKNV-0618 was used to prepare an oil-based FKC vaccine as previously described (20). Briefly, ISKNV-0618 was propagated in MFF-1 cells (36). The virus titer was measured by TCID_50_ values (47). The virus suspension was inactivated by formalin with a final concentration of 0.1% (vol/vol) at 4°C for 10 days. Then, an antigenic dose of the inactivated virus was adapted with sterile PBS and emulsified with injectable white oil (ExxonMobil; catalog no. 8042-47-5). The final dose of the vaccine was adjusted to 10^6.5^ TCID_50_/0.1 mL. The vaccine was kept at 4°C until use. Ninety spotted seabass with an average body weight of 30 g and 90 mandarin fish with an average body weight of about 50 g were administered by intraperitoneal injection with 0.1 mL of the ISKNV-FKC vaccine. At 21 dpv, both immunized fish were divided into three groups and challenged by intraperitoneal injection with SBIV-VP13, SBIV-V12, and ISKNV-0618, respectively, with 30 spotted seabass and 30 mandarin fish for each virus. Similar challenges were operated on both equal untreated spotted seabass and mandarin fish control groups. Mortality was monitored daily for 20 days. Statistical analysis was conducted by Fisher’s exact test. The relative percentage survival (RPS) was calculated by the following formula: RPS = [1 − (mortality of immunized fish/mortality of unimmunized control fish)] × 100%.

### Histopathology, IHC, and IFA.

Tissues from moribund fish were picked out and fixed with alcohol-formaldehyde (AFA) for hematoxylin and eosin (H&E) staining or with 4% paraformaldehyde for immunohistochemistry (IHC) and immunofluorescence detection (IFA) as previously described (7). IHC was performed using rabbit anti-ISKNV-VP23 serum (1:2,000) as the primary antibody (48) and horseradish peroxidase (HRP)-labeled goat anti-rabbit IgG as the secondary antibody according to the supersensitive IHC detection system kit (mouse/rabbit) (Bioworld Technology, Inc.). IFA was conducted using a mouse anti-ISKNV-VP23 monoclonal antibody (Zhang W et al., unpublished data) and a rabbit anti-ISKNV-VP101 as primary antibodies (7, 49). AlexaFluor488-conjugated (green fluorescent) goat anti-rabbit IgG and AlexaFluor555-conjugated (red fluorescent) goat anti-mouse IgG were used as the secondary antibodies (Invitrogen, USA). The nuclei were stained by 4′,6-diamidino-2-phenylindole (DAPI) (Abcam, China). Sections were observed under a confocal laser scanning immunofluorescence microscope (Leica SP8; Germany).

### TEM.

The spleens from diseased mandarin fish were collected for TEM analysis as in our previous studies (7, 36). The ultrathin sections were stained with uranyl acetate-lead citrate and examined under a Jeol JEM-1400 electron microscope (Japan).

### Data availability.

Whole-genome sequences of RISV-I type SBIV-VP13 and RSIV-II type SBIV-V12 have been deposited in the GenBank database with accession numbers ON740976 and ON743042, respectively.

## References

[1] Eaton HE, Ring BA, Brunetti CR. 2010. The genomic diversity and phylogenetic relationship in the family *Iridoviridae*. Viruses 2:1458–1475. doi:10.3390/v2071458. 21994690 PMC3185713

[2] Chinchar VG, Hick P, Ince IA, Jancovich JK, Marschang R, Qin Q, Subramaniam K, Waltzek TB, Whittington R, Williams T, Zhang Q-Y, ICTV Report Consortium. 2017. ICTV virus taxonomy profile: *Iridoviridae*. J Gen Virol 98:890–891. doi:10.1099/jgv.0.000818. 28555546 PMC5656800

[3] World Organisation for Animal Health (OIE). 2021. Red sea bream iridoviral disease. *In* Manual of diagnostic tests for aquatic animals. World Organisation for Animal Health, Paris, France.

[4] Zhang W, Deng H, Fu Y, Fu W, Weng S, He J, Dong C. 2023. Production and characterization of monoclonal antibodies against mandarinfish ranavirus and first identification of pyloric caecum as the major target tissue. J Fish Dis 46:189–199. doi:10.1111/jfd.13733. 36441809

[5] Kawato Y, Mekata T, Inada M, Ito T. 2021. Application of environmental DNA for monitoring red sea bream iridovirus at a fish farm. Microbiol Spectr 9:e00796-21. doi:10.1128/Spectrum.00796-21. 34704786 PMC8549737

[6] Kurita J, Nakajima K. 2012. Review: megalocytiviruses. Viruses 4:521–538. doi:10.3390/v4040521. 22590684 PMC3347321

[7] Zhu ZM, Duan C, Li Y, Huang CL, Weng SP, He JG, Dong CF. 2021. Pathogenicity and histopathology of infectious spleen and kidney necrosis virus genotype II (ISKNV-II) recovering from mass mortality of farmed Asian seabass, *Lates calcarifer*, in Southern China. Aquaculture 534:736326. doi:10.1016/j.aquaculture.2020.736326.

[8] Shinmoto H, Taniguchi K, Ikawa T, Kawai K, Oshima S. 2009. Phenotypic diversity of infectious red sea bream iridovirus isolates from cultured fish in Japan. Appl Environ Microbiol 75:3535–3541. doi:10.1128/AEM.02255-08. 19346349 PMC2687316

[9] Fu Y, Li Y, Fu W, Su H, Zhang L, Huang C, Weng S, Yu F, He J, Dong C. 2021. Scale drop disease virus associated yellowfin seabream (*Acanthopagrus latus*) ascites diseases, Zhuhai, Guangdong, southern China: the first description. Viruses 13:1617. doi:10.3390/v13081617. 34452481 PMC8402775

[10] de Groof A, Guelen L, Deijs M, van der Wal Y, Miyata M, Ng KS, van Grinsven L, Simmelink B, Biermann Y, Grisez L, van Lent J, de Ronde A, Chang SF, Schrier C, van der Hoek L. 2015. A novel virus causes scale drop disease in *Lates calcarifer*. PLoS Pathog 11:e1005074. doi:10.1371/journal.ppat.1005074. 26252390 PMC4529248

[11] Tsai JM, Huang SL, Yang CD. 2020. PCR detection and phylogenetic analysis of megalocytivirus isolates in farmed giant sea perch *Lates calcarifer* in southern Taiwan. Viruses 12:681. doi:10.3390/v12060681. 32599850 PMC7354458

[12] Ishihara H, Harakawa S, Kawakami H, Yoshii K, Murase N, Yamada H, Fukuda Y, Nozaki R, Kawato S, Koiwai K, Hirono I, Kondo H. 2022. Whole-genome analysis of red sea bream iridovirus spread in 2021 in Japan provided epidemiological and viral traits insight. J Fish Dis 45:1593–1597. doi:10.1111/jfd.13690. 35862188

[13] He JG, Deng M, Weng SP, Li Z, Zhou SY, Long QX, Wang XZ, Chan SM. 2001. Complete genome analysis of the mandarin fish infectious spleen and kidney necrosis iridovirus. Virology 291:126–139. doi:10.1006/viro.2001.1208. 11878882

[14] Puneeth TG, Baliga P, Girisha SK, Shekar M, Nithin MS, Suresh T, Naveen Kumar BT. 2021. Complete genome analysis of a red seabream iridovirus (RSIV) isolated from Asian seabass (*Lates calcarifer*) in India. Virus Res 291:198199. doi:10.1016/j.virusres.2020.198199. 33080247

[15] Kayansamruaj P, Soontara C, Dong HT, Phiwsaiya K, Senapin S. 2020. Draft genome sequence of scale drop disease virus (SDDV) retrieved from metagenomic investigation of infected barramundi, *Lates calcarifer* (Bloch, 1790). J Fish Dis 43:1287–1298. doi:10.1111/jfd.13240. 32829517

[16] Fu Y, Li Y, Chen J, Yu F, Liu X, Fu W, Pan H, Li W, Weng S, He J, Dong C. 2023. A mandarinfish *Siniperca chuatsi* infection and vaccination model for SDDV and efficacy evaluation of the formalin-killed cell vaccine in yellowfin seabream *Acanthopagrus latus*. Aquaculture 570:739428. doi:10.1016/j.aquaculture.2023.739428.

[17] Inouye K, Yamano K, Maeno Y, Nakajima K, Matsuoka M, Wada Y, Sorimachi M. 1992. Iridovirus infection in farmed red sea bream. Fish Pathol 27:19–27. doi:10.3147/jsfp.27.19.

[18] Kawakami H, Nakajima K. 2002. Marine cultured fish species with red sea bream iridovirus disease confirmed from 1996 to 2000. Fish Pathol 37:45–47. doi:10.3147/jsfp.37.45.

[19] Lopez-Porras A, Morales JA, Alvarado G, Koda SA, Camus A, Subramaniam K, Waltzek TB, Soto E. 2018. Red seabream iridovirus associated with cultured Florida pompano Trachinotus carolinus mortality in Central America. Dis Aquat Organ 130:109–115. doi:10.3354/dao03267. 30198486

[20] Dong Y, Weng S, He J, Dong C. 2013. Field trial tests of FKC vaccines against RSIV genotype Megalocytivirus in cage-cultured mandarin fish (*Siniperca chuatsi*) in an inland reservoir. Fish Shellfish Immunol 35:1598–1603. doi:10.1016/j.fsi.2013.09.005. 24035751

[21] Go J, Whittington R. 2019. Experimental transmission of infectious spleen and kidney necrosis virus (ISKNV) from freshwater ornamental fish to silver sweep *Scorpis lineolata*, an Australian marine fish. Dis Aquat Organ 137:1–21. doi:10.3354/dao03422. 31777395

[22] Ramirez-Paredes JG, Paley RK, Hunt W, Feist SW, Stone DM, Field TR, Haydon DJ, Ziddah PA, Nkansa M, Guilder J, Gray J, Duodu S, Pecku EK, Awuni JA, Wallis TS, Verner-Jeffreys DW. 2021. First detection of infectious spleen and kidney necrosis virus (ISKNV) associated with massive mortalities in farmed tilapia in Africa. Transbound Emerg Dis 68:1550–1563. doi:10.1111/tbed.13825. 32920975 PMC8246855

[23] Figueiredo HCP, Tavares GC, Dorella FA, Rosa JCC, Marcelino SAC, Pierezan F, Pereira FL. 2022. First report of infectious spleen and kidney necrosis virus in Nile tilapia in Brazil. Transbounding Emerging Dis 69:3008–3015. doi:10.1111/tbed.14217. 34223695

[24] Wang YQ, Lu L, Weng SP, Huang JN, Chan SM, He JG. 2007. Molecular epidemiology and phylogenetic analysis of a marine fish infectious spleen and kidney necrosis virus-like (ISKNV-like) virus. Arch Virol 152:763–773. doi:10.1007/s00705-006-0870-4. 17131065

[25] Johan CAC, Zainathan SC. 2020. Megalocytiviruses in ornamental fish: a review. Vet World 13:2565–2577. doi:10.14202/vetworld.2020.2565-2577. 33363355 PMC7750215

[26] Fu X, Li N, Liu L, Lin Q, Wang F, Lai Y, Jiang H, Pan H, Shi C, Wu S. 2011. Genotype and host range analysis of infectious spleen and kidney necrosis virus (ISKNV). Virus Genes 42:97–109. doi:10.1007/s11262-010-0552-x. 21107672

[27] Shi C-Y, Wang Y-G, Yang S-L, Jie H, Wang Q-Y. 2004. The first report of an iridovirus-like agent infection infarmed turbot, *Scophthalmus maximus*, in China. Aquaculture 236:11–25. doi:10.1016/j.aquaculture.2003.11.007.

[28] Go J, Waltzek TB, Subramaniam K, Yun SC, Groff JM, Anderson IG, Chong R, Shirley I, Schuh JC, Handlinger JH, Tweedie A, Whittington RJ. 2016. Detection of infectious spleen and kidney necrosis virus (ISKNV) and turbot reddish body iridovirus (TRBIV) from archival ornamental fish samples. Dis Aquat Organ 122:105–123. doi:10.3354/dao03068. 28000602

[29] Oh MJ, Kitamura SI, Kim WS, Park MK, Jung SJ, Miyadai T, Ohtani M. 2006. Susceptibility of marine fish species to a megalocytivirus, turbot iridovirus, isolated from turbot, *Psetta maximus* (L.). J Fish Dis 29:415–421. doi:10.1111/j.1365-2761.2006.00734.x. 16866925

[30] Kim WS, Oh MJ, Jung SJ, Kim YJ, Kitamura S. 2005. Characterization of an iridovirus detected from cultured turbot *Scophthalmus maximus* in Korea. Dis Aquat Organ 64:175–180. doi:10.3354/dao064175. 15918481

[31] Nakajima K, Maeno Y, Kurita J, Inui Y. 1997. Vaccination against red sea bream iridoviral disease in red sea bream. Fish Pathol 32:205–209. doi:10.3147/jsfp.32.205.

[32] Dong C, Xiong X, Luo Y, Weng S, Wang Q, He J. 2013. Efficacy of a formalin-killed cell vaccine against infectious spleen and kidney necrosis virus (ISKNV) and immunoproteomic analysis of its major immunogenic proteins. Vet Microbiol 162:419–428. doi:10.1016/j.vetmic.2012.10.026. 23177910

[33] Zhang WF, Duan C, Zhang HT, Weng SP, He JG, Dong CF. 2020. Widespread outbreaks of the emerging mandarinfish ranavirus (MRV) both in natural and ISKNV-FKC vaccinated mandarinfish *Siniperca chuatsi* in Guangdong, South China, 2017. Aquaculture 520:734989. doi:10.1016/j.aquaculture.2020.734989.

[34] Dong HT, Jitrakorn S, Kayansamruaj P, Pirarat N, Rodkhum C, Rattanarojpong T, Senapin S, Saksmerprome V. 2017. Infectious spleen and kidney necrosis disease (ISKND) outbreaks in farmed barramundi (*Lates calcarifer*) in Vietnam. Fish Shellfish Immunol 68:65–73. doi:10.1016/j.fsi.2017.06.054. 28663128

[35] Imajoh M, Ikawa T, Oshima S. 2007. Characterization of a new fibroblast cell line from a tail fin of red sea bream, *Pagrus major*, and phylogenetic relationships of a recent RSIV isolate in Japan. Virus Res 126:45–52. doi:10.1016/j.virusres.2006.12.020. 17335926

[36] Dong C, Weng S, Shi X, Xu X, Shi N, He J. 2008. Development of a mandarin fish *Siniperca chuatsi* fry cell line suitable for the study of infectious spleen and kidney necrosis virus (ISKNV). Virus Res 135:273–281. doi:10.1016/j.virusres.2008.04.004. 18485510

[37] Dong C, Weng S, Luo Y, Huang M, Ai H, Yin Z, He J. 2010. A new marine megalocytivirus from spotted knifejaw, *Oplegnathus punctatus*, and its pathogenicity to freshwater mandarinfish, Siniperca chuatsi. Virus Res 147:98–106. doi:10.1016/j.virusres.2009.10.016. 19895861

[38] Eaton HE, Metcalf J, Penny E, Tcherepanov V, Upton C, Brunetti CR. 2007. Comparative genomic analysis of the family *Iridoviridae*: re-annotating and defining the core set of iridovirus genes. Virol J 4:11. doi:10.1186/1743-422X-4-11. 17239238 PMC1783846

[39] Lü L, Zhou SY, Chen C, Weng SP, Chan SM, He JG. 2005. Complete genome sequence analysis of an iridovirus isolated from the orange-spotted grouper, *Epinephelus coioides*. Virology 339:81–100. doi:10.1016/j.virol.2005.05.021. 15964605

[40] Chen XH, Lin KB, Wang XW. 2003. Outbreaks of an iridovirus disease in maricultured large yellow croaker, *Larimichthys crocea* (Richardson), in China. J Fish Dis 26:615–619. doi:10.1046/j.1365-2761.2003.00494.x. 14653319

[41] Ni SZ, Wang YJ, Hu JB, Shi J, Xu Y, Zhou SM, Li JJ, Hong BH, Qian D. 2021. Identification, histopathology, and phylogenetic analysis of an iridovirus from cultivated silver pomfret in Zhejiang Province, East China. Aquaculture 530:735619. doi:10.1016/j.aquaculture.2020.735619.

[42] Zheng ZY, Chi HS, Liu XD, Yang XX, Chen XX, Pan Y, Gong H. 2023. A new embryonic cell line YCE1 from large yellow croaker (*Larimichthys crocea*) and its susceptibility to large yellow croaker iridovirus. Aquaculture 565:739079. doi:10.1016/j.aquaculture.2022.739079.

[43] Zhang W, Dong Y, Weng S, Dong C. 2019. Immunological prevention of an emerging red sea bream iridovirus [RSIV] in cage-cultured spotted mandarinfish *Siniperca scherzeri* in Dandong, Northeast China. J Animal Vet Sci 1:1025.

[44] Jung MH, Lee J, Jung SJ. 2016. Low pathogenicity of flounder iridovirus (FLIV) and the absence of cross-protection between FLIV and rock bream iridovirus. J Fish Dis 39:1325–1333. doi:10.1111/jfd.12459. 27009694

[45] Fu Y, Li Y, Zhang W, Fu W, Li W, Zhu Z, Weng S, He J, Dong C. 2023. Effectively protecting Asian seabass *Lates calcarifer* from ISKNV-I, ISKNV-II, RSIV-II and SDDV by an inactivated ISKNV-I and SDDV bivalent vaccine. Aquaculture 566:739218. doi:10.1016/j.aquaculture.2022.739218.

[46] Lehwark P, Greiner S. 2019. GB2sequin-a file converter preparing custom GenBank files for database submission. Genomics 111:759–761. doi:10.1016/j.ygeno.2018.05.003. 29842948

[47] Reed LJ, Muench H. 1938. A simple method of estimating fifty percent endpoints. Am J Hyg 27:493–497. doi:10.1093/oxfordjournals.aje.a118408.

[48] Xu X, Weng S, Lin T, Tang J, Huang L, Wang J, Yu X, Lu L, Huang Z, He J. 2010. VP23R of infectious spleen and kidney necrosis virus mediates formation of virus-mock basement membrane to provide attaching sites for lymphatic endothelial cells. J Virol 84:11866–11875. doi:10.1128/JVI.00990-10. 20810728 PMC2977902

[49] Dong C, Xiong XP, Shuang F, Weng SP, Zhang J, Zhang Y, Luo YW, He JG. 2011. Global landscape of structural proteins of infectious spleen and kidney necrosis virus. J Virol 85:2869–2877. doi:10.1128/JVI.01444-10. 21209107 PMC3067969

